# Supplementary material for: Genome-Wide Analysis and Function Prediction of Long Noncoding RNAs in Sheep Pituitary Gland Associated with Sexual Maturation
Source: Genes (Basel). 2020 Mar 17;11(3):320. doi: 10.3390/genes11030320 (PMC7140784; doi:10.3390/genes11030320)
Supplement: Supplementary file 1 [file genes-11-00320-s001.zip › Table S1.docx]

Table S1 The list of primer sequences for lncRNAs and mRNAs.

| Gene symbol | Primer Sequence | Product size (bp) |
| --- | --- | --- |
| *GAPDH* | F: GTCAAGGCAGAGAACGGGAA  R: GGTTCACGCCCATCACAAAC | 232 |
| *XM_012095561.3* | F: TTCTCTCGCATGGGACCCTA  R: TCGTCCAGGTTGAAGTGAGC | 233 |
| *TCONS_00060829* | F: TCTTGTCGGCATGAAGGGAC  R: CTCGCTCCAAAGAGGCTTCA | 176 |
| *TCONS_00034478* | F: TTGAGAGCATGCCCCTTCTG  R: CGGTACCGCAAGATACCTCC | 142 |
| *XM_004002368.4* | F: TGTCAACGGCAAGCTGTTTC  R: GGCGTGGACATCCTGGTATT | 104 |
| *TCONS_00019332* | F: AAACCCACCTAGCGCAGAAA  R: CGGTAAAGTGGCCGGGTATT | 151 |
| *XR_003586084.1* | F: GCTCGTCCAGGATCTCTCAC  R: AGGCCTTATCAGAAACGAGG | 104 |
| *XR_003588753.1* | F: CTGAGCCTCTGCTTACGAGG  R: CTCTCACCAACGCCGAGATT | 236 |
| *XR_003589149.1* | F: CGTACCTGTTCTCTCCAGCC  R: CGCCATACCTGGCTTAGTGT | 172 |
| *FSH* | F: GGGGCATCAGCTTCAGTTCT  R: GAGCTTGGTAACTGCCACCT | 107 |
| *LH* | F: GGCTACTGCCTCAGCATGAA  R: GGTGGGCATGGGAGATTGA | 292 |
| Gene symbol | **TCONS_00066406 siRNA sequence (5’-3’)** | **Target sites** |
| siRNA-NC | sense: UUCUCCGAACGUGUCACGUTT  antisense: ACGUGACACGUUCGGAGAATT | Negative control |
| siRNA- TCONS_00066406 | sense: GCAUGUAGGGUGACAAAUATT  antisense: UAUUUGUCACCCUACAUGCTT | 1785 |
