# Supplementary material for: Genome-Wide Analysis and Function Prediction of Long Noncoding RNAs in Sheep Pituitary Gland Associated with Sexual Maturation
Source: Genes (Basel). 2020 Mar 17;11(3):320. doi: 10.3390/genes11030320 (PMC7140784; doi:10.3390/genes11030320)
Supplement: Supplementary file 1 [file genes-11-00320-s001.zip › Table S2.docx]

**Table S2. Statistical results of reads in each pituitary samples**

| Sample | Total Reads | Unmapped Reads | Unique Mapped Reads | Multiple Mapped reads | Mapping Ratio |
| --- | --- | --- | --- | --- | --- |
| TM1 | 65372520 | 4910609 (7.51%) | 59276005 (90.67%) | 1185906 (1.81%) | 92.49% |
| TM2 | 65030068 | 4640502 (7.14%) | 59107004 (90.89%) | 1282562 (1.97%) | 92.86% |
| TM3 | 83600252 | 5920280 (7.08%) | 76252840 (91.21%) | 1427132 (1.71%) | 92.92% |
| NM1 | 73552520 | 5100660 (6.93%) | 67321100 (91.53%) | 1130760 (1.54%) | 93.07% |
| NM2 | 76893112 | 5488471 (7.14%) | 70175057 (91.26%) | 1229584 (1.60%) | 92.86% |
| NM3 | 58651926 | 3480175 (5.93%) | 53659603 (91.49%) | 1512148 (2.58%) | 94.07% |

1) TM1, TM2, TM3 and NM1, NM2, NM3 represent the 3-month-old (immature) and 9-month-old (mature) sheep pituitary sample, respectively.

2) Total Reads: The number of clean reads.

3) Unmapped Reads: The number of sequences not matched to the genome.

4) Unique Mapped Reads: The number of sequences that had unique positions mapped to the reference sequence.

5) Multiple Mapped Reads: The number of sequences that had multiple positions mapped to the reference sequence.

6) Mapping Ratio: The ratio of mapped reads to total reads.
