# Supplementary material for: Genome-Wide Analysis and Function Prediction of Long Noncoding RNAs in Sheep Pituitary Gland Associated with Sexual Maturation
Source: Genes (Basel). 2020 Mar 17;11(3):320. doi: 10.3390/genes11030320 (PMC7140784; doi:10.3390/genes11030320)
Supplement: Supplementary file 1 [file genes-11-00320-s001.zip › Table S3.docx]

**Table S3. The GO enrichment analysis of DE mRNAs.**

| Function | GO terms | Gene number | UP-regulated | DOWN-regulated | p value |
| --- | --- | --- | --- | --- | --- |
| Hormone synthesis and regulation | response to hormone | 28 | 19 | 9 | 0.004354 |
|  | response to steroid hormone | 13 | 8 | 5 | 0.009713 |
|  | negative regulation of intracellular steroid hormone receptor signaling pathway | 5 | 2 | 3 | 0.020998 |
|  | regulation of intracellular steroid hormone receptor signaling pathway | 6 | 3 | 3 | 0.035261 |
|  | hormone-mediated signaling pathway | 10 | 8 | 2 | 0.049685 |
|  | intracellular steroid hormone receptor signaling pathway | 6 | 4 | 2 | 0.059886 |
|  | steroid hormone mediated signaling pathway | 6 | 4 | 2 | 0.059886 |
|  | cellular response to steroid hormone stimulus | 6 | 4 | 2 | 0.068861 |
|  | steroid metabolic process | 6 | 1 | 5 | 0.156328 |
|  | regulation of steroid metabolic process | 3 | 1 | 2 | 0.333417 |
|  | steroid biosynthetic process | 2 | 0 | 2 | 0.34677 |
|  | hormone secretion | 2 | 1 | 1 | 0.359172 |
|  | positive regulation of intracellular steroid hormone receptor signaling pathway | 1 | 1 | 0 | 0.40445 |
|  | regulation of secretion | 10 | 3 | 7 | 0.411945 |
|  | endocrine hormone secretion | 1 | 0 | 1 | 0.463123 |
|  | regulation of hormone levels | 5 | 3 | 2 | 0.472786 |
|  | hormone metabolic process | 1 | 0 | 1 | 0.54837 |
|  | regulation of hormone secretion | 2 | 1 | 1 | 0.63574 |
| Growth | growth | 20 | 10 | 10 | 0.016486 |
|  | tissue development | 45 | 23 | 22 | 0.030739 |
|  | cell proliferation | 23 | 9 | 14 | 0.040928 |
|  | response to growth factor | 11 | 5 | 6 | 0.047896 |
|  | animal organ development | 69 | 35 | 34 | 0.058599 |
|  | organ maturation | 1 | 0 | 1 | 0.066741 |
|  | cellular response to growth factor stimulus | 8 | 4 | 4 | 0.086159 |
|  | negative regulation of cellular response to growth factor stimulus | 1 | 0 | 1 | 0.129036 |
|  | organ growth | 1 | 1 | 0 | 0.267213 |
|  | regulation of multicellular organism growth | 2 | 1 | 1 | 0.520627 |
|  | regulation of growth | 12 | 7 | 5 | 0.563437 |
|  | negative regulation of growth | 1 | 1 | 0 | 0.721778 |
|  | developmental growth | 3 | 1 | 2 | 0.768882 |
|  | regulation of developmental growth | 2 | 1 | 1 | 0.872635 |
| Reproduction | positive regulation of reproductive process | 3 | 1 | 2 | 0.074064 |
|  | male gamete generation | 11 | 4 | 7 | 0.294436 |
|  | multicellular organism reproduction | 14 | 7 | 7 | 0.35067 |
|  | multicellular organismal reproductive process | 13 | 6 | 7 | 0.398003 |
|  | reproductive process | 27 | 13 | 14 | 0.412347 |
|  | reproduction | 27 | 13 | 14 | 0.420603 |
|  | negative regulation of Wnt signaling pathway | 5 | 3 | 2 | 0.472786 |
|  | regulation of Notch signaling pathway | 2 | 1 | 1 | 0.488288 |
|  | canonical Wnt signaling pathway | 1 | 0 | 1 | 0.498996 |
|  | regulation of Wnt signaling pathway | 7 | 3 | 4 | 0.510612 |
|  | reproductive behavior | 1 | 1 | 0 | 0.516026 |
|  | multicellular organism reproductive behavior | 1 | 1 | 0 | 0.516026 |
|  | multi-organism reproductive process | 14 | 5 | 9 | 0.61273 |
|  | Wnt signaling pathway | 2 | 1 | 1 | 0.660906 |
|  | sexual reproduction | 12 | 3 | 9 | 0.697153 |
|  | positive regulation of Wnt signaling pathway | 2 | 0 | 2 | 0.824359 |
|  | developmental process involved in reproduction | 8 | 3 | 5 | 0.89518 |
|  | gonad development | 1 | 0 | 1 | 0.939481 |
|  | cellular process involved in reproduction in multicellular organism | 1 | 1 | 0 | 0.941547 |
|  | development of primary sexual characteristics | 1 | 0 | 1 | 0.941547 |
|  | reproductive structure development | 2 | 0 | 2 | 0.943315 |
|  | reproductive system development | 2 | 0 | 2 | 0.943315 |
