# Supplementary figures and images for: Genome-Wide Analysis and Function Prediction of Long Noncoding RNAs in Sheep Pituitary Gland Associated with Sexual Maturation
Source: Genes (Basel). 2020 Mar 17;11(3):320. doi: 10.3390/genes11030320 (PMC7140784; doi:10.3390/genes11030320)

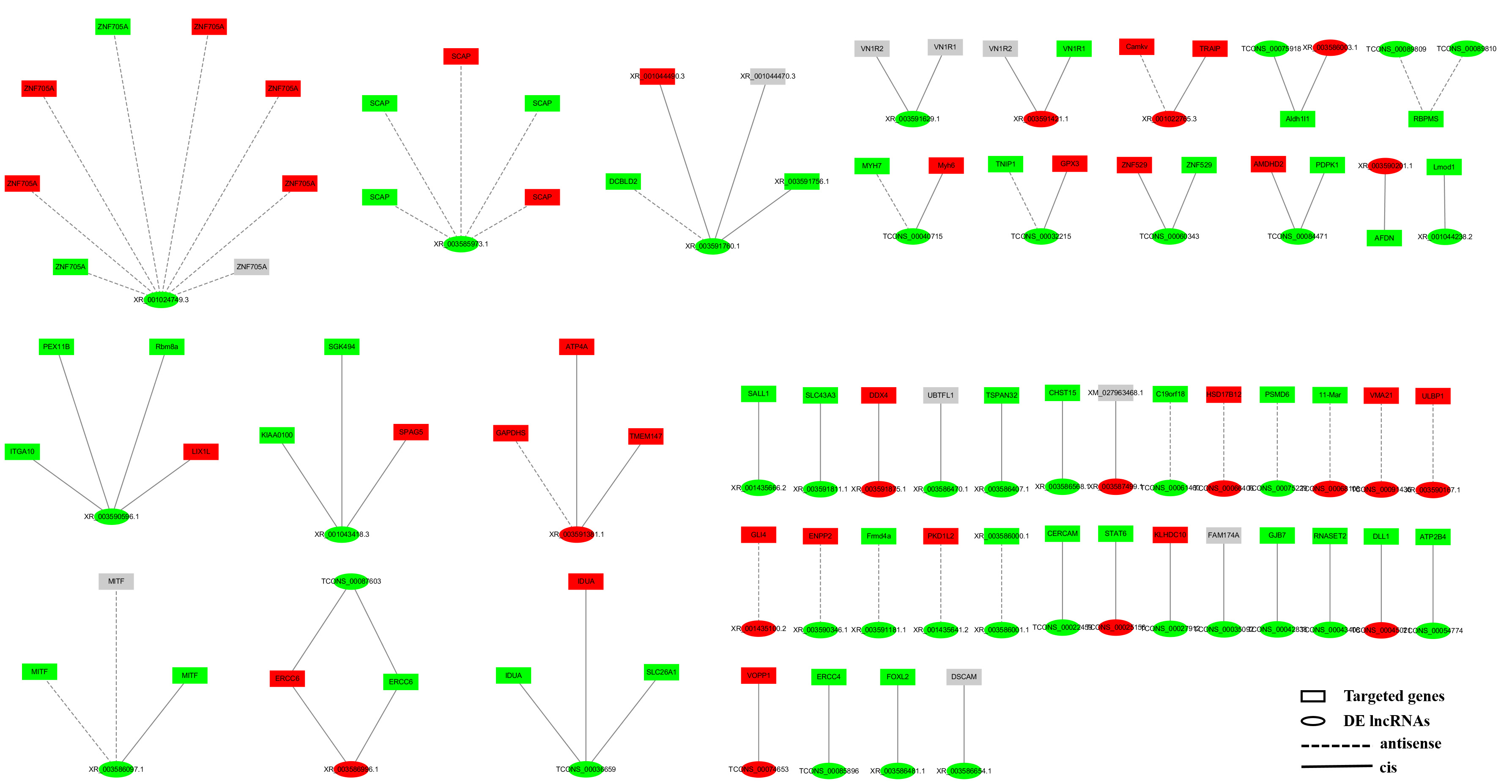

Supplement: Supplementary file 1 [file genes-11-00320-s001.zip › Fig S1.jpg]
